# Thermo-ring basis for heat unfolding-induced inactivation in TRPV1

**DOI:** 10.21203/rs.3.rs-3280283/v2

**Published:** 2024-05-09

**Authors:** Guangyu Wang

**Affiliations:** 1 Department of Physiology and Membrane Biology, University of California School of Medicine, Davis, CA 95616, USA; 2 Department of Drug Research and Development, Institute of Biophysical Medico-chemistry, Reno, NV 89523, USA

**Keywords:** cyclization against decyclization, desensitization, grid thermodynamics, lipid, non-covalent interaction, noxious heat detection, systemic thermal instability, thermoring, thermosensitivity, threshold

## Abstract

Transient receptor potential vanilloid-1 (TRPV1) is a capsaicin receptor and employs the use-dependent desensitization to protect mammals from noxious heat damage in response to repeated or constant heat stimuli. However, the underlying structural factor or motif has not been resolved precisely. In this computational study, the graph theory-based grid thermodynamic model was used to reveal how the temperature-dependent noncovalent interactions as identified in the 3D structures of rat TRPV1 could develop a well-organized fluidic grid-like mesh network, featuring various topological grids constrained as the thermo-rings that range in size from the biggest to the smallest to govern distinct structural and functional traits of the channel in response to varying temperature degrees. Following the findings that the heat unfolding of three specific biggest grids, one in the closed state and two in the open state, was respectively responsible for the reversible activation at 43 °C and thermal inactivation from 56 °C to 61 °C, a random smaller grid was further identified for the irreversible inactivation and the relevant use-dependent desensitization from the pre-open closed state between 43 °C and 61 °C. Thus, these two distinct inactivation pathways of TRPV1 may be involved in protecting mammals against noxious heat damages.

Transient receptor potential (TRP) vanilloid-1 (TRPV1) in mammals is a capsaicin receptor and characterized by the intriguing temperature-dependent inactivation in response to a heat stimulus. Following the activation at a start temperature threshold 43 °C, its activity peaks at 56 °C and then declines until 61 °C^1–3^. Meanwhile, in response to repeated or constant heat stimuli, this thermosensitive channel exhibits the overall irreversible use-dependent desensitization between 43 °C and 61 °C^1, 3, 4^, which is independent of activation by heat or capsaicin and necessary to prevent cell swelling/death of TRPV1-expressing cells and thus to lower the risk of scald injury in mammals^4^. Since the rundown-evoked decrease in current noises of TRPV1 in liposomes is similar to the Zn^2+^-induced inactivation of externally cysteine-engineered cystic fibrosis transmembrane conductance regulator (CFTR)^5–6^, different inactivation pathways in distinct temperature ranges may be involved in TRPV1 gating to protect mammals against noxious heat damages.

On the other hand, as (TRP) vanilloid-3 (TRPV3) displays the use-dependent sensitization upon repeated heat stimuli^7–13^, an exchange of the C-terminus between rat TRPV1 (rTRPV1) and mouse TRPV3 (mTRPV3) was used to eliminate the use-dependent heat desensitization in rTRPV1^14.^. Following that report, the thermosensitive dynamic interaction between the N- and C-termini (segments 1–433 and 688–839, respectively) of mouse TRPV1 (mTRPV1) rather than platypus (*Orni-thorhynchus anatinus*) TRPV1 (pTRPV1) has also been indicated to trigger a conformational rearrangement in the outer pore, causing inactivation^4^. Although the temperature-dependent 3D cryo-electron microscopy (cryo-EM) structures of rTRPV1 with phosphatidylinositol (PI) or capsaicin (Cap) bound at low and high temperatures have been available^15^, much less is known about the precise structural factors or motifs to differentiate temperature-dependent inactivation pathways.

Recently, graph theory has been employed to investigate the temperature-dependent noncovalent interactions as found in the 3D structures of various globular and integral membrane proteins including thermosensitive TRPV1 and TRPV3 and TRP melastatin-8 (TRPM8)^16–21^. Of special interest, once these interactions assemble into a well-organized fluidic grid-like mesh network, the topological grids can be constrained as specific thermal rings or thermo-rings that range in size from the biggest to the smallest grids to match featured functional data from chimeric and single point mutation studies, and thereby validating the specific thermo-ring structure. For example, once three specific biggest grids have been identified in closed and open states of rTRPV1, their heat unfolding can attribute to matched structural and functional traits of the channel in response to the following temperature degrees.

First, in the presence of PI at the vanilloid site, the biggest Grid_13_ at the interface between the pre-S1 domain and the voltage sensor-like domain (VSLD) of rTRPV1 may control the Y401-R499 cation- interaction for the normal start activity temperature 43 °C^19^. Alternatively, the biggest Grid_14_ at the pre-S1/VSLD/TRP interface near PI of human TRPV1 (hTRPV1) may govern the E406-K504 (E405-K504 in rTRPV1) salt bridge for the similar start activity temperature 41 °C^19^. In support of this notion, the T406D (T407 in hTRPV1) mutation in this biggest Grid_13_ has been reported to slow down the heat activation of rTRPV1^22^, and phosphorylation of nearby S502 by protein kinase C enhances the thermal sensitivity of TRPV1^23–24^. On the other hand, the pre-S1 segment 358–434 of mTRPV3 has been first shown to mediate the temperature threshold (T_th_) and sensitivity Q_10_^2^, and later the insertion of valine at position 412 of mTRPV3 (405 in rTRPV1 or 406 in hTRPV1) induces the formation of a similar thermo-ring Grid_17_ at the pre-S1/VSLD interface to control the R416-D519 salt bridge near the phosphatidylcholine (PC) pocket at the corresponding vanilloid site, decreasing the first start activity temperature from 52 °C to 42 °C^13, 20^. Similarly, the swapping of the pre-S1 domain from rTRPV1 into rTRPV2, hTRPV2, or mTRPV4 alters their heat sensation to a different extent^25^. Notably, the replacement of the segment ^365^KD^366^ of rTRPV2 with the equivalent ^405^ET^406^ of rTRPV1 decreases the activation threshold T_th_ from 52 °C to 46 °C^25^.

Second, the biggest Grid_9’_ in the open state of PI-free rTRPV1 has a 9-residue size to control D576-T685 and F580-L678 bridges at the S5/S6 interface for the maximal activity temperature 61 °C via the thermo-ring from D576 to F580, L678, T685 and back to D576^3, 19^. Since any mutation like R579E/A or D576N/R or L678A in rTRPV1 or T680A in mTRPV3 (T685 in rTRPV1) in this Grid_9’_ leaves the channel non-functional or insensitive to heat^26–28^, any thermostable perturbation around this theromo-ring may bring about the partial thermal inactivation of the ion conduction pathway from the open state, evoking flickering opening^16, 29^.

Finally, another biggest Grid_9_ in the open state of PI-free rTRPV1 also has a 9-residue size to control the stimulatory R557-E570 H-bond at the VSLD/S4-S5 linker/TRP interfaces for the maximal activity at 56 °C via the thermo-ring from R557 to E570, Q560, W697, R701, Q700, and back to R557^2, 3, 19^. When the temperature raises above 56 °C, the weakened R557-E570 salt bridge may also allow thermal inactivation from the open state^3^. In agreement with this notion, the R557E or E570L mutant has no function, or mutants R557A/L and E570A or nearby M572A are insensitive to heat^15. 26, 30^, or substitution of I696, W697 and R701 by alanine severely compromises the heat-dependent activation^31^. In that regard, the biggest Grid_9_ in the open state may be responsible for the optimal activity temperature 56 °C.

Taken together, these three specific thermo-rings are responsible for reversible gating transitions at specific activity temperatures. In this study, such a graphical analysis was further used to reveal a random smaller thermo-ring at the pre-S1/TRP interface for the irreversible heat-unfolding-induced inactivation from a pre-open closed state and the relevant use-dependent desensitization from 43 °C to 61 °C, along with a matched structural and functional inactivation thermo-sensitivity and a significant decrease in the systematic thermal instability^3^.

## Results

### Cold unfolding destabilized PI-free rTRPV1 along with a lower melting threshold

**Figure 1.**
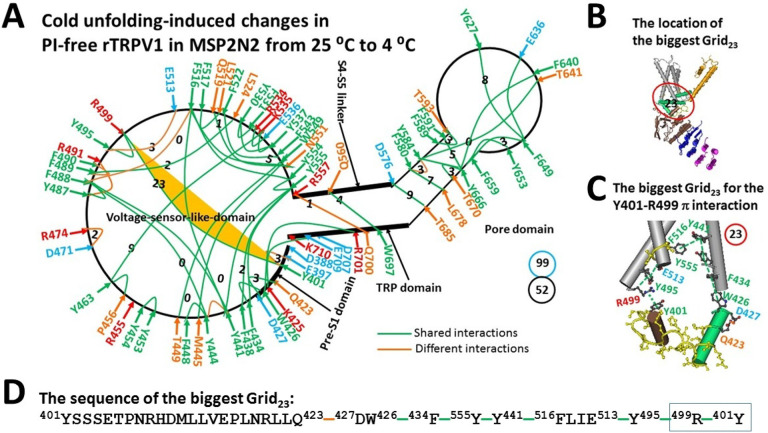
The grid-like noncovalently interacting mesh network along the PI-dependent minimal gating pathway of capsaicin-bound rTRPV1 in the closed state at 4 °C. (**A)** The topological grids in the systemic fluidic grid-like mesh network. The cryo-EM structure of one subunit in closed rTRPV1 with Cap bound at 4 °C (PDB ID, 7LPA) is used for the model. The pore domain, the S4-S5 linker, the TRP domain, the VSLD and the pre-S1 domain are indicated in black arrows. The shared and different noncovalent interactions between two closed states at 4 °C and at 25 °C are colored in green and orange, respectively. The constrained grid sizes required to control the relevant noncovalent interactions are labeled in black numbers. The Y401-R499 bridge in the biggest Grid_23_ is highlighted. The total grid sizes and grid size-controlled noncovalent interactions along the defined PI-dependent minimal gating pathway from D388 to K710 are shown in the cyan and black circles, respectively. (**B)** The location of the biggest Grid_23_ at the pre-S1/VSLD interface. (**C)** The structure of the biggest Grid_23_ with a 23-residue size at the pre-S1/VSLD interface to control the R499-Y401 interaction. The grid size is shown in a red circle. (**D)** The sequence of the biggest Grid_23_ to control the R499-Y401 interaction in the blue box.

When temperature decreased from 25 °C to 4 °C, the K688-E692 salt bridge and the Q691-N695 and Q560-K694 H-bonds at the TRP/S6 interface as found in PI-free closed state at 25 °C were broken (Fig.1A),^19^ possibly because interfacial ordered water molecules may form an H-bonding network with the charged residues such as K688 and E692. As a result, PI-free rTRPV1 at 4 °C had nine new noncovalent interactions along the previously defined PI-dependent minimal gating pathway from D388 in the pre-S1 domain to K710 in the TRP domain^19^. In addition to R557-Q700 and Y487-R491 and D471-R474 H-bonds, most of them were interactions between E397-Y401, Y441-Y444, F488-F516, F489-F490, Y495-R499, Y495-E513, or F580-Y584 pairs (Fig. 1A, Table S1). When the total non-covalent interactions and the total grid sizes decreased from 54 and 89 to 52 and 99, respectively (Fig. 1A), the systematic thermal instability (T_i_) increased from 1.65 to 1.90 (Table 1). Of special note, the biggest Grid_23_ was present to control the same Y401-R499 interaction at the pre-S1/VSLD interface (Fig. 1A). It had a 23-residue size via the shortest path from Y401 to Q423, D427, W426, F434, Y555, Y441, F516, E513, Y495, R499 and back to Y401 (Fig. 1B–D). When two equivalent H-bonds sealed it, the calculated T_m_ decreased from 32 to 28 °C so that the channel was still closed at 4 °C (Table 1)^15, 19^.

### Heat unfolding induced an inactivated state between 43 to 61 °C

**Figure 2.**
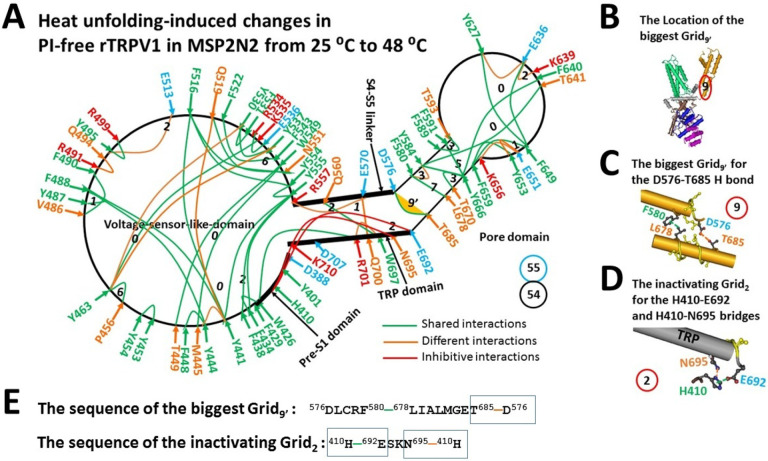
The grid-like noncovalently interacting mesh network along the PI-dependent minimal gating pathway of PI-free rTRPV1 in an inactivated state at 48 °C. (**A)** The topologocal grids in the systemic fluidic grid-like mesh network. The cryo-EM structure of one subunit of rTRPV1 with Cap bound at 48 °C for 30 s (PDB ID, 7LPD) is used as a model for an inactivated state. The pore domain, the S4-S5 linker, the TRP domain, the VSLD and the pre-S1 domain are indicated in black arrows. The shared and different noncovalent interactions between the closed state at 25 °C and the inactivated state at 48 °C are colored in green and orange, respectively. Of special note, the inhibitive noncovalent interactions are colored in red. The constaint grid sizes required to control the relevant noncovalent interactions are labeled in black numbers. The D576-T685 H-bond in the biggest Grid_9’_ is highlighted in yellow. The total grid sizes and grid size-controlled non-covalent interactions along the defined PI-dependent minimal gating pathway from D388 to K710 are shown in the cyan and black circles, respectively. (**B)** The location of the biggest Grid_9’_. (**C)** The structure of the biggest Grid_9’_ at the S5/S6 interface to control the D576-T685 H-bond. The grid size is shown in a red circle. (**D)** The inactivating Grid_2_ to govern the E692-H410-N695 bridges. The grid size is shown in a red circle. (**E)** The sequences of the biggest Grid_9’_ and the inactivating Grid_2_ with 9- and 2-residue sizes to control the D576-T685 and E692-H410-N695 bridges in the blue rectangles, respectively.

When temperature increased from 25 °C to 48 °C to unfold the Y401-R499 interaction at the pre-S1/VSLD interface,^19^ fifteen new non-covalent interactions were observed in the 7LPD class for 30 s (Fig. 2A). They included one E651-K656 salt bridge, seven -interacting pairs such as H410-E692, Y441-Y444, Y463-Y530, Y495-E513, F580-Y584, Y627-E636 and W697-R701, and 8 H-bonding pairs such as H410-N695, Y441-Q519, Y463-Y537, R491-Q494, R557-E570, E570-Q700, E636-K639 and E651-Y653 (Fig. 2A, Table S2). In this case, the stimulatory R557-E570 H-bond as identified in the biggest Grid_9_ of the open state at 48 °C was governed by the smaller Grid_1_ in this class via the shortest path or thermo-ring from R557 to E570, Q700, R701, W426, F434, Y555 and back to R557 (Fig. 2A). Once five equivalent H-bonds sealed it, the calculated T_m_ was up to 102 °C. Thus, Grid_1_ could not be spontaneously rearranged to the biggest Grid_9_ for channel opening at 48 °C.

On the other hand, the biggest Grid_9’_ in the pore domain of the open state also appeared in this 7LPD class after 30 s (Figs. 2A–C & 2E)^19^. For 2.5 equivalent H-bonds to seal this Grid_9’_, the calculated T_m_ was still about 61 °C (Table 1). In that case, such a high temperature could prevent Grid_9’_ from melting below 61 °C to allow a thermo-ring rearrangement from Grid_1_ to Grid_9_ to open the channel^19^. What is more, as this biggest Grid_9’_ is a necessary anchor to keep the intact active pore domain of all the gating states including the open state of TRPV1^19^, it is not allowed to melt. Thereby, this 7LPD class could not be a pre-open closed state but an inactivated state below 61 °C. In agreement with the above proposal, when the open state served as a control, the calculated apparent mean structural thermo-sensitivity (Ω_10_) was about −5.28, which was similar to the measured Q_10_ of −5.09 for the heat inactivation (Table 1)^3^. Furthermore, when the total non-covalent interactions and the total grid sizes along the PI-dependent minimal gating pathway of the 7LPD class were 54 and 55, respectively (Fig.2A), the calculated systematic thermal instability (T_i_) was actually 1.02, which was much smaller than that of the open state (1.65) as reported previously (Table 1)^19^. Hence, this 7LPD class was very stable, favoring rundown in response to repeated heat stimuli.^3, 5^

### Identification of a structural motif for the heat inactivation between 43 and 61 °C

**Figure 3.**
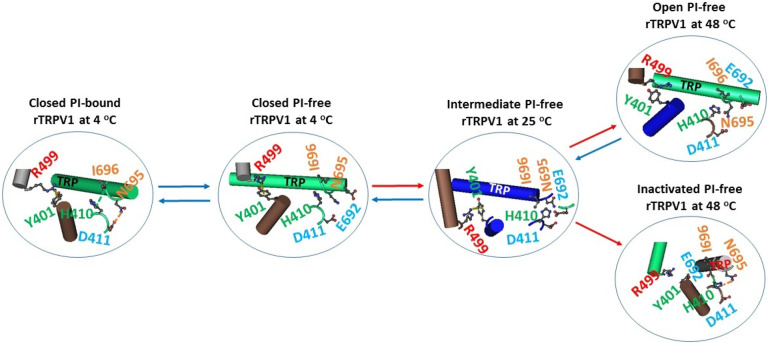
Noncovalent interactions at the VSLD/pre-S1/TRP interfaces along the temperature-dependent gating transitions of rTRPV1. The models are based on closed PI-bound at 4 °C (PDB ID, 7LP9), closed Cap-bound at 4 °C (PDB ID, 7LPA), intermediate RTX-bound at 25 °C (PDB ID, 7RQX), open Cap-bound at 48 °C (PDB ID, 7LPE), and inactivated Cap-bound at 48 °C (PDB ID, 7LPD).

When compared with the open state^19^, in addition to the common W426-R701 interaction, an H410-E692 interaction and an H410-N695 H-bond were unique in the inactivated state, forming a smaller Grid_2_. It had a 2-residue size to control those two inhibitive bridges via the shortest path or thermos-ring from H410 to E692, N695 and back to H410 (Fig. 2A & 2D–E, Table S2). With two equivalent H-bonds sealing it, the calculated T_m_ was about 70 °C. Therefore, it could not spontaneously melt at 48 °C to reverse the inactivated state to an open one unless the biggest Grid_9’_ was thermally unfolded at 61 °C. Further structural comparison showed that both intermediate and open states of rTRPV1 with resiniferatoxin (RTx) bound at 25 or 48 °C had the only W426-R701 interaction at the pre-S1/TRP interface (Fig. 3, Table 2)^32^. Therefore, the smaller Grid_2_ at the pre-S1/TRP interface was the structural motif for the inactivation between 41 °C and 61 °C^3^.

### Inactivating Grid_2_ in PI-free rTRPV1 at 48 °C was created by heat rather than capsaicin

It is interesting that the smallest Grid_0_ also appeared at the same pre-S1/TRP interface of closed PI-bound rTRPV1 at 48 °C (PDB ID, 7LPC). It had a 0-residue size to control the H410-I696 interaction and the D411-N695 H-bond via the shortest path or thermo-ring from H410 to I696, N695, D411 and back to H410.^19^ Since it was similar to the smaller Grid_2_, it may also act as an inhibitive motif, preventing channel opening at 48 °C.^15^ Furthermore, such an inhibitive motif was also present in PI-bound rTRPV1 at 4 °C but disappeared together with the inhibitive Grid_2_ in Cap-bound rTRPV1 at 4 °C (Figs. 1A, 2A, 3). In that regard, the inhibitive Grid_2_ was factually formed by the heat stimulus rather than capsaicin. This finding was consistent with the notion that capsaicin and heat desensitize TRPV1 on a distinct structural basis^14^.

### The broken Y401-R499 cation- interaction was required for heat-evoked channel opening and inactivation

A previous study showed that the Y401-R499 cation- interaction serves as a critical thermostable lock at the pre-S1/VSLD interface for channel closure in PI-bound rTRPV1 at 48 °C and PI-free rTRPV1 at 25 °C^19^. Further investigation demonstrated that such a lock also existed in the closed state of PI-bound or free rTRPV1 at 4 °C, the intermediate state of PI-free rTRPV1 at 25 °C. However, it was opened in the open and inactivated states of PI-free rTRPV1 at 48 °C (Fig.3). In this regard, the Y401-R499 cation- interaction needed to be broken for heat-evoked channel opening and inactivation.

### The E692-H410-N695 bridges induced a strong inhibitive E570-Q700 H-bond at the active S4-S5 linker/TRP interface

**Figure 4.**
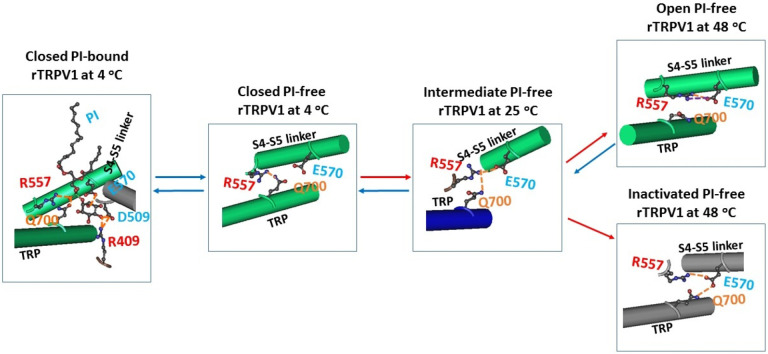
Noncovalent interactions at the S4-S5 linker/TRP/pre-S1 interfaces along the temperature-dependent gating transitions of rTRPV1. The models are based on closed PI-bound at 4 °C (PDB ID, 7LP9), closed Cap-bound at 4 °C (PDB ID, 7LPA), intermediate RTX-bound at 25 °C (PDB ID, 7RQX), open Cap-bound at 48 °C (PDB ID, 7LPE), and inactivated Cap-bound at 48 °C (PDB ID, 7LPD).

Although the Q560-W697 interaction at the active S4-S5 linker/TRP interface was shared by the resting closed states at 4 °C and 25 °C, the open state at 48 °C and the inactivated state at 48 °C (Figs. 1A & 2A),^19^ different noncovalent interactions at such an active center were observed along the gating transitions (Fig.4). In the resting closed PI-bound rTRPV1 at 4 °C, PI linked R557 and E570 in the S4-S5 linker, Q700 in the TRP domain, R409 in the pre-S1 domain and D509 in the VSLD together via H-bonds, forming a strong inhibitive triangle with R557, E570 and Q700. When Cap competed off the PI lipid at 4 °C, R557 only H-bonded with Q700 via the side chains. When the temperature increased to 25 °C, R557 continued to H-bond with E570 via the side chains in the intermediate state of PI-free rTRPV1. When the temperature increased to 48 °C, the channel was open with the R557-Q700 H-bond disrupted but the R557-E570 H-bond enhanced by the additional salt bridge between them. The similar cases were also found in the open state of RTX-bound open states at 25 and 48 °C (Table 2).^32^ In contrast, in the inactivated state at the same temperature of 48 °C, the R557-Q700 H-bond moved to the E570-Q700 H-bond. Thus, once the E692-H410-N695 bridges were formed upon heat unfolding, the strong inhibitive E570-Q700 H-bond was also born at the S4-S5 linker/TRP interface for the final channel inactivation (Fig.4).

### Heat unfolding-induced E692-H410-N695 bridges were random for the irreversible inactivation

**Figure 5.**
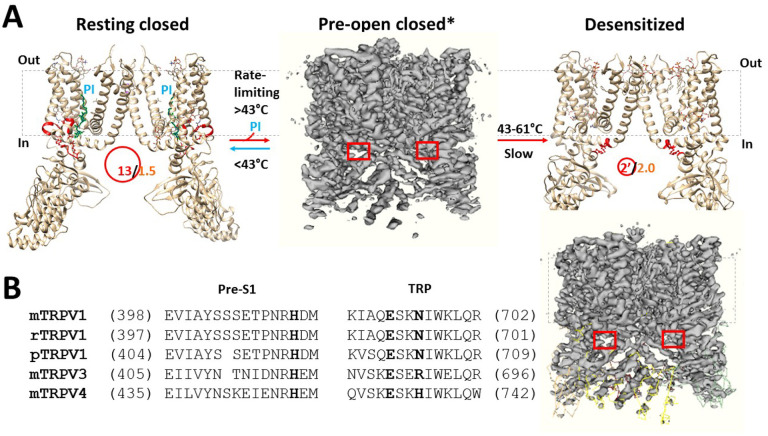
Tentative model for the irreversible inactivation of rTRPV1 from the pre-open closed state upon the heat unfolding of the biggest grid. (**A**) The homo-tetrameric cryo-EM structures of rTRPV1 in the resting closed, inactivated, and pre-open closed states (PDB ID: 7LP9, 7LPD/EMD-23478 and EMD-23477, respectively) are used for the models. For convenience, only two opposite subunits are shown for resting closed and inactivated states. The dashed rectangles are the membrane areas. In the presence of the PI lipid (blue highlighted) at the active capsaicin site, rTRPV1 has 1.5 equivalent H-bonds (orange) to seal the biggest Grid_13_ (red) at the VSLD/pre-S1 interface so that rTRPV1 is closed below a threshold 43 °C. When the temperature increases above 43 °C to remove the PI lipid from the active vanilloid site, a random smaller Grid_2_ (red) at the pre-S1/TRP interface is absent in the pre-open closed state but present in the inactivated state. The lower systematic thermal instability T_i_ (1.02) in the inactivated state may allow rTRPV1 to bypass the open state and to inactivate slowly from the pre-open closed state between 43 °C and 61 °C in an irreversable manner. (**B)** The sequence alignment of TRPV1–4 alongside the pre-S1 domain and the TRP domain.

In addition to the 7LPD class at 48 °C for 30 s, the major class at 48 °C for 10 s (EMD 23477) has been reported as a pre-open closed state.^15^ Further comparison of cryo-EM density maps uncovered that although these two classes shared a similar overall structure, the major class 48 °C for 10 s (EMD 23477), like the intermediate state of RTX-bound rTRPV1 at 25 °C (7RQX), had not the density blob to correspond to the E692-H410-N695 bridges and thus could serve as an intermediate of the closed-to-open transition (Fig. 5). In contrast, the side chain of H410 had the broad lower density blob in the 7LPD class at 48 °C for 30 s (Fig. 5). Thus, high mobility of the side chain of H410 may allow the different orientations to determine whether the inhibitive E692-H410-N695 bridges were born or not for channel inactivation or opening, respectively. In other words, heat unfolding-induced E692-H410-N695 bridges were factually random. Once they were created, the strong inhibitive E570-Q700 H-bond could be induced at the S4-S5 linker/TRP interface for the final irreversible inactivation (Fig. 4). More importantly, unlike cold unfolding in PI-free rTRPV1 at 4 °C (Fig. 1A), heat unfolding at higher temperature may further dehydrate the pre-S1/TRP interface to stabilize the smaller Grid_2_.^4^ That may be the reason for rapid inactivation above 47 °C after prolonged repeated heat stimuli .^3,5^

## Discussion

The TRPV1 bio-thermometer takes a critical role in detecting noxious heat and protecting human or animal bodies from heat damage^1, 33–35^. Recently, a graph theory approach has been developed to elucidate its temperature sensation^19^. Once the networks of temperature-dependent non-covalent interactions such as hydrogen bonds, salt bridges and -interactions are available, well-organized fluidic-like grids of various sizes have been comprehensively constrained and defined as the thermo-rings. Since each thermo-ring attributes to distinct structural and functional traits of the channel in response to various temperatures, the thermo-rings along the gating pathways are pivotal for temperature sensitivity. Following the identification of three specific biggest grids as the thermo-rings in closed and open states for the activity temperatures from the starting 43 °C to the optimal 56 °C and then to the ending 61 °C^19^, this graphical method was further exploited to identify a random smaller thermo-ring at the pre-S1/TRP interface for the heat unfolding-induced irreversible inactivation from 43 °C to 61 °C and the relevant use-dependent desensitization upon constant or repeated noxious heat stimuli^2, 3, 14^.

Previous structural data of rTRPV1 indicated that the 7LPD class at 48 °C after 30 s shares the similar 3D structure with the major class at 48 °C after 10 s ^15^. This similarity is reminiscent of the similar overall structures between resting and inactivated states in the Kv4 channel^36–38^ Therefore, like the proposed inactivation from a pre-open closed state for Kv4 channels to desensitize to the voltage response, once the major class at 48 °C after 10 s serves as the pre-open closed state, it would also be followed by the 7LPD class at 48 °C after 30 s as the inactivated state between 43 °C and 61 °C for several reasons (Figs. 2A & 4A). First, for the same amount of the TRPV1 sample with 30 μM capsaicin, the initial particle images of the major class at 48 °C after 10 s are the twice as more as those of the 7LPD class or the open state at 48 °C when the channel activity is maximal after 30 s (PDB ID, 7LPE);^4–5, 15^ Second, the biggest Grid_9’_ had the calculated T_m_ of 61 °C to allow the inactivated state to survive in the activity temperature range from 43 °C to 61 °C (Figs. 2A–C & 2E)^2–4^; Third, the inactivating Grid_2_ at the pre-S1/TRP interface had a T_m_ of 70 °C. It was not present in any other gating states including the intermediate state at 25 °C once the PI lipid was released from the vanilloid site (Fig. 3, Table 2)^15, 19, 32^. Fourth, The inhibitive Y401-R49 interactions still appeared in the intermediate of PI-free rTRPV1 at 25 °C but disappeared in the 7LPD class at 48 °C for 30 s (Fig. 3, Table 2); Fifth, the inactivated state had a much lower systematic thermal instability (T_i_) of 1.02 than that of the closed or open state in PI-free rTRPV1 (T_i_, ~1.65) (Table 1). Hence, although the inactivation rate is slower than the activation rate at similar temperature of 45 °C for 30 s ^3–5, 14^, the thermo-stable inactivated state, once yielded, facilitated the rundown between 43 °C and 61 °C.

A previous study demonstrated that a random formation of the C612-C619 disulfide bond in response to short heat stimuli is responsible for the heat sensitization of TRPV3. 20 In this study, once the Y401-R499 cation- interaction in the pre-open closed state was broken (Fig. 2A, Table 2), the side chain of H410 could orientate randomly in different ways, either for channel opening in the absence of the smaller Grid_2_ (PDB ID, 7LPE) or for channel inactivation in the presence of the smaller Grid_2_ as a “thermostable lock” (PDB ID, 7LPD), preventing the channel transition to any of the functional states. It had an H-bond between H410-N695 and a lone pair- interaction between H410 and E692 via the side chains (Fig. 3). In direct agreement with this proposal, the final particle images in the 7LPD class had a broad lower density blob of the side chain of H410 (Fig. 5A).^15^ On the other hand, since these inhibitive bridges were located at the intracellular water/protein interface, dehydration at higher temperature may enhance these inhibitive bridges at the pre-S1/TRP interface,^4^ accelerating the inactivation above 47 °C after prolonged heat stimuli.^3,5^ In line with this notion, unfolded proteins become more compact when the temperature is raised but disordered at low temperature^39^, more protein-water H-bonds were found in the cold-denatured state of yeast frataxin than in the hot-denatured state^40^, and the strong electrostatic interactions between ions and water molecules stabilize yeast frataxin^41^. In this case, although the inhibitive E692-H410-N695 bridges were random, once formed upon heat unfolding of the Y401-R499 bridge and enhanced by elevated temperature, the inhibitive E570-Q700 H-bond was also generated at the S4-S5 linker/TRP interface, prohibiting gating transition to any of the other functional states unless supersaturated capsaicin bridges T550 and E570 together to disrupt the E570-Q700 H-bond .^4, 42^ More importantly, the inactivation may further trigger heat-induced protein aggregation in an irreversible manner^5^. Taken together, all these factors may promote the irreversible heat unfolding-induced inactivation of rTRPV1 between 43 °C and 61 °C and the relevant use-dependent desensitization in response to constant or repeated heat stimuli.^3–5^

It is interesting that the sequence alignment reveals that the missing serine between E404 (E397 in rTRPV1) and H416 (H410 in rTRPV1) of pTRPV1 or the substitution of N695 in rTRPV3 by R690 in mTRPV3 may prohibit the formation of the inactivating Grid_2_ at the pre-S1/TRP interface (Fig. 5B)^43, 44^. This difference may account for the absence of the use-dependent desensitization when the N- and C-terminal domains of rTRPV1 are replaced by those homologous residues of pTRPV1 or mTRPV3.^4,14^ On the other hand, rTRPV1 or mTRPV1 shares similar pre-S1 and TRP domains with mTRPV4 (Fig. 5B). Thus, both mTRPV4 and rTRPV1 may use the same or similar inactivation pathway for the use-dependent heat desensitization^45, 46^. In addition, unlike HEK293 cell-expressing rTRPV1, oocytes expressing rTRPV1 exhibits minimal desensitization of heat-evoked responses^47^, possibly by weakening the inactivating E692-H410-N695 bridges in the different membrane system. Finally, it has been reported that the association between the intracellular helix-loop-helix (HLH) near pre-S1 domain and the TRP domain helix favors the faster inactivation kinetics observed in mammalian TRPV6 channels^48^, and E692 is required for TRPV1 activity^49^. All these reports are consistent with the regulatory role of the pre-S1-TRP noncovalent interaction in mammalian TRPV1. Further structural and functional measurements are invited to test the role of the random E692-H410-N695 bridges or smaller Grid_2_ in the irreversible heat inactivation and the relevant use-dependent desensitization and maximal P_o_ limitation between 43 °C and 61 °C.

## Conclusion

The graph theory-based grid thermodynamic model has been successfully used to define the specific structural motifs for activation and inactivation of biological globular and integral membrane proteins including two classes of fructose aldolases and thermosensitive TRPV1 and TRPV3 and TRPM8. While the melting of the specific biggest grid near the active site is generally required for reversible heat activation, the thermal unfolding of the specific active site or the nearby anchor grid is necessary for reversible thermal inactivation. Regarding temperature sensation of TRPV1, two specific biggest grids in the open state of rTRPV1 have been recently identified. Their heat unfolding results in reversible thermal inactivation from the open state, underlying the formation of optimal and maximal activity temperatures. In this computational study, a random smaller inhibitive grid near the lower gate was further identified to be responsible for the irreversible heat inactivation from the pre-open closed state and the relevant use-dependent desensitization in a whole activity temperature range. Collectively, these two different thermal inactivation pathways may underlie the intricate mechanisms of temperature sensation within the TRPV1 biothermometer.

## Computational Methods

### Data mining resources

Several 3D cryo-EM structures of rTRPV1 in MSP2N2 in different gating states were used to construct the systematic fluidic grid-like noncovalent interactin mesh networks. They included the closed state with PI bound at 4 °C (PDB ID, 7LP9, model resolution = 2.63 Å) and 48 °C (PDB ID, 7LPC, model resolution = 3.06 Å), the closed state with capsaicin (Cap) bound at 4 °C (PDB ID, 7LPA, model resolution = 3.37 Å), the pre-open closed state with Cap bound at 48 °C for 10 s (EMD ID, 23477, model resolution = 3.70 Å) and the putatively inactivated state with Cap bound at 48 °C for 30 s (EMD 23478; PDB ID, 7LPD, model resolution = 3.55 Å)^15^. Meanwhile, the open state with Cap bound at 48 °C (PDB ID, 7LPE, model resolution = 3.72 Å), the closed state with Cap bound at 25 °C (PDB ID, 7LPB, model resolution = 3.54 Å), and the intermediate state with resiniferatoxin (RTx) bound at 25 °C (PDB ID, 7RQX, model resolution = 3.36 Å) and the open state with RTX bound at 25 °C (PDB ID, 7RQY, model resolution = 3.04 Å) and 48 °C (PDB ID, 7RQZ, model resolution = 3.32 Å) were also used as important controls to identify a random structrual motif for the irreversible heat inactivation of rTRPV1^15, 32^.

### Definition of the necessary PI-dependent minimal gating pathway

The phosphatidylinositol (PI)-dependent minimal gating pathway from D388 in the pre-S1 domain to K710 in the TRP domain has been successfully defined in rTRPV1 to identify the specific structural motifs for reversible heat activation with the matched temperature thresholds and sensitivity and systematic thermal instability (T_i_)^19^. Therefore, it was also used in this study to identify a random structural motif for the irreversible heat inactivation between 43 °C and 61 °C. The flexible segments beyond this PI-dependent minimal gating pathway were not considered because of the low resolution^15, 32^.

### Standards to filter non-covalent interactions

UCSF Chimera was used to visualize any possible stereo-selective or regio-selective inter-domain diagonal and intra-domain lateral noncovalent interactions along the defined PI-dependent minimal gating pathway of rTRPV1. They included salt-bridges, lone pair/CH/cation- interactions and H-bonds between paired amino acid side chains. The same strict and consistent standard definition of noncovalent interactions as described and examined previously was employed for the results to be reproduced well with a high sensitivity^16–21^. Details of specific cutoff distances and interaction angles for the different noncovalent interactions were also shown in the online Supplementary Information (Tables S1, S2). Of special note, only the same group of protein structures obtained in the same model membrane systems were used for comparison unless they were unavailable. Furthermore, momentary fluctuation-induced perturbations in the noncovalent interactions during protein dynamics were not considered. In this way, approximately at least 50 different noncovalent interactions were identified along the PI-dependent minimal gating pathway from D388 to K710 on each protomer.

### Mapping topological grids by using graph theory

The same protocol as descibed and examined previously was used to map the topological grids in this study.^16–21^ First, the identified noncovalent interactions and linked residues along the PI-dependent minimal gating pathway from D388 to K710 (black line) were respectively projected as edges and node arrows on the systematic fluidic mesh network. The shared and different noncovalent interactions between the closed state at 25 °C and the closed state at 4 °C or the inactivated state at 48 °C were colored in green and orange, respectively. Meanwhile, the inhibitive noncovalent interactions were presented in red. Second, all the grids were then contoured on the map after their thermo-ring sizes were constrained as the minimal number of the total free side chains of residues that did not involve any non-covalent interction in a grid. The grid size was constrained with graph theory and the Floyd-Warshall algorithm^50^. In this way, the smallest size of a grid or a thermo-ring was available from the shortest reverse path between two ends of a linked non-covalent interaction because the direct path was always zero. For example, in the grid-like biochemical reaction mesh network of Fig.2A, a direct path length from Y627 and E636 was zero because there was an H-bond between them. However, there was another shortest reverse path from E636 to F649 and back to Y627 via two interactions. Because nothing but these three residues was involved in the non-covalent interactions, the grid size was zero. Third, once each noncovalent interaction was tracked with a grid size, the unshared sizes were then marked in black numbers on the network map. Finally, a grid with an x-residue was denoted as Grid_x._ Meanwhile, the total noncovalent interactions and the total grid sizes along the defined PI-dependent minimal gating pathway of one subunit were calculated and shown in black and cyan circles beside the mesh network map, respectively, in favor of calculating the systematic thermal instability (T_i_) and the structural thermo-sensitivity (Ω_10_).

### Calculation of the temperature threshold of rTRPV1

The melting temperature threshold (T_m_) for thermal unfolding of a given grid was calculated by using the same equation as examined by the structural data of several proteins such as class I and II fructose aldolases aldolases and TRPV1 and TRPV3 and TRPM8^16–21^:

(1) $$T_{m}\left({}^{\circ}C\right)=34+\left(n-2\right)\times 10+(20-S_{max})\times 2$$

where, n is the total number of basic H-bonds ( ~1 kcal/mol for each) energetically equivalent to the noncovalent interactions controlled by the given grid, and S_max_ is the size of the given grid. Accordingly, a decrease in the grid size or an increase in equivalent H-bonds will raise the grid’s heat capacity.

### Assessment of the grid-based systemic thermal instability (T_i_)

The systematic thermal instability (T_i_) along the defined PI-dependent minimal gating pathway was calculated by using the same equation as described and empirically examined previously^16–21^:

(2) $$T_{i}=S/N$$

where, S and N are the total grid sizes and the total non-covalent interactions along the same PI-dependent minimal gating pathway of one subunit in a given gating state. On the ground of this definition, the lower T_i_ means the less compact conformational entropy in the system.

### Evaluation of the systematic temperature sensitivity of rTRPV1

For enthalpy-driven inactivation of TRPV1 from the pre-open closed state within 10 °C as a result of the broken biggest grid, if the chemical potential of a grid is theoretically defined as the maximal potential for equivalent residues in the grid to form the tightest -hairpin with the smallest loop via non-covalent interactions^51^, the grid-based structural thermo-sensitivity (Ω_10_) of a single ion channel for the heat inactivation could be defined and calculated using the following equations:

(3) $$\Omega_{10}=-{\left[(S_{i}-S_{o})E/2\right]}^{\left(Hi/Ho\right)}=-\;{\left[(S_{i}-S_{o})E/2\right]}^{\left[(ENi/(ENo)\right]}=-\;{\left[(S_{i}-S_{o})E/2\right]}^{\left(Ni/No\right)}$$

where, along the same PI-dependent gating pathway of one subunit, N_i_ and N_o_ are the total non-covalent interactions, H_i_ and H_o_ are the total enthalpy included in them, and S_i_ and S_o_ are the total grid sizes in the inactivated and open states, respectively. E is the energy intensity of a noncovalent interaction in a range of 0.5–3 kcal/mol. Usually, E is 1 kcal/mol. Thus, Ω_10_ factually reflects a thermo-evoked change in the total chemical potential of grids upon a thermo-evoked change in the total enthalpy included in the noncovalent interactions apparently from an open state to an inactivated state along the same PI-dependent minimal gating pathway of one subunit.

For convenient comparison, the functional thermo-sensitivity (Q_10_) of a single ion channel for the heat inactivation was calculated using the following equation:

(4) $$Q_{10}=-{\left(X_{2}/X_{1}\right)}^{10/(T2-T1)}$$

where, X_1_ and X_2_ are relative decreases in channel activity obtained at temperatures T1 and T2 (measured in kelvin), respectively.

## Figures and Tables

**Table 1:** Grid thermodynamic model-based new parameters induced by cold- and heat-unfolding of rTRPV1. The comparative parameters are highlighted in bold.

Construct	rTRPV1 in MSP2N2				
PDB ID	7LPE		7LPA	7LPB	7LPD
Lipid PI at the capsaicin site	free		free	free	free
Sampling temperature, ° C	48		4	25	48
Gating state	open		closed	closed	inactivated
Name of the biggest grid	Grid_9_	Grid_9’_	Grid_23_	Grid_21_	Grid_9’_
Biggest grid size (S_max_)	9	9	23	21	9
Equivalent basic H-bonds to control S_max_	2.0	2.5	2.0	2.0	2.5
Total non-covalent interactions	43		52	54	54
Total grid sizes, a.a./atoms	71		99	89	55
Systematic thermal instability (T_i_)	1.65		1.90	1.65	1.02
Calculated T_m_, °C	**56**	**61**	**28**	**32**	**61**
Measured threshold T_th_, °C	**56**	**61**	**<40**	**<40**	**61**
Calculated Ω_10_, _min_ at E = 0.5 kJ/mol					−3.03
Calculated Ω_10_, _mean_ at E = 1.0 kJ/mol					−**5.28**
Calculated Ω _10_, _max_ at E = 3.0 kJ/mol					−12.7
Measured Q10					−**5.09**
References for T_th_ and Q_10_	[2, 3, 19]	[3, 19]	[3, 15]	[3, 15]	[3]

**Table 2.** Inter-domain noncovalent interactions in different gating states of rTRPV1.

PDB ID	7LPD	7LPE	7LPC	7LPB	7LPA	7LP9	7RQX	7RQY	7RQZ
ligand	Cap	Cap	PI	Cap	Cap	PI	RTx	RTx	RTx
Sampling T, °C	48	48	48	25	4	4	25	25	48
Gating state	inactivated	open	closed	closed	closed	closed	intermediate	open	open
R557-E570	+	+	−	−	−	−	+	+	+
R557-(PI)-Q700	−	−	+	−	+	+	+	−	−
E570-(PI)-Q700	+	−	+	−	−	+	−	−	−
E397-K710	−	−	+	−	−	+	−	−	−
Y401-R499	−	−	+	+	+	+	+	−	−
H410-E692	+	−	−	−	−	−	−	−	−
H410/-N695/I696	+	−	+	−	−	+	−	−	−
W426-R701	+	+	+	+	+	+	+	+	+

Note: C terminal region, N687-K838; N-terminal region, M1-R432. The R557-E570 bridge was a control at the S4-S5 linker/VSLD interface.

## Data Availability

All data generated or analysed during this study are included in this published article and Supplementary Information.

## Abbreviations

Cap: capsaicin
cryo-EM: cryo-electron microscopy
CFTR: cystic fibrosis transmembrane conductance regulator
Ω_10_: structural thermosensitivity
Q_10_: functional thermo-sensitivity
PC: phosphatidylcholine
PI: phosphatidylinositol
RTx: resiniferatoxin
T_i_: systematic thermal instability
T_m_: melting temperature threshold
T_th_: temperature threshold
TRP: transient receptor potential
TRPV1: TRP vanilloid-1
TRPVi: (i=1; 2, 3, 4, 5, 6)
hTRPV1: human TRPV1
hTRPV2: human TRPV2
mTRPV1: mouse TRPV1
mTRPV3: mouse TRPV3
mTRPV4: mouse TRPV4
pTRPV1: platypus TRPV1
rTRPV1: rat TRPV1
rTRPV2: rat TRPV2
TRPM8: TRP melastatin-8
VSLD: voltage-sensor-like domain
